# No evidence that same‐language subtitles improve children's reading fluency

**DOI:** 10.1111/bjop.70066

**Published:** 2026-03-08

**Authors:** Anastasiya Lopukhina, Holly Cooper, Cheng‐Yu Hsieh, Walter J. B. van Heuven, Kathleen Rastle

**Affiliations:** ^1^ Department of Psychology Royal Holloway, University of London Egham UK; ^2^ School of Psychology University of Nottingham Nottingham UK

**Keywords:** closed captions, eye‐tracking, intervention, primary‐school children, reading, same‐language subtitles

## Abstract

High‐profile campaigns globally have argued that same‐language television subtitles may help children improve their reading. In this intervention study, we tested the causal hypothesis that exposure to subtitles improves children's reading fluency. We tested 127 British children in Years 2 and 3 before and after a 6‐week home‐based intervention, in which children were randomly assigned to an experimental group that watched television with English subtitles or a control group that watched television without subtitles. Children's reading fluency was assessed via two standard tests of reading fluency and their engagement with subtitles while watching videos, measured with eye‐tracking. Results showed that both groups improved their reading fluency over 6 weeks. Crucially, there was no evidence that subtitle exposure (of around 66 hr on average) drove an improvement in reading fluency, or that greater subtitle exposure provided any additional benefit. We conclude that same‐language subtitles are unlikely to improve children's reading fluency. However, the primary school reading curriculum has a positive impact on reading fluency that can be observed over a relatively short time‐scale.

Reading is a fundamental skill that enables people to access knowledge, education, social benefits and public services. However, most children in low‐ and middle‐income countries fail to learn to read (World Bank, 2019), and the same is true for around 6% of children in high‐income countries (Mullis et al., 2023). Some children never receive the instruction necessary to develop foundational reading skills (Crawford et al., 2025). Others may not get enough reading practice due to a lack of engagement in independent reading or limited access to books (Cole et al., 2022). For example, in the United Kingdom, only about one‐third of children report that they enjoy reading in their free time, and just one in five report reading daily outside of school (National Literacy Trust, 2024).

Three high‐profile campaigns – *Turn on the Subtitles* in the United Kingdom (https://turnonthesubtitles.org/), *PlanetRead* in India (https://www.planetread.org/) and *Switch On Read On* globally (https://switchonreadon.org/) – have proposed an innovative solution to this global literacy challenge. These campaigns argue that turning on same‐language subtitles while children watch television can dramatically improve their reading skills. The *Turn on the Subtitles* campaign claims that the benefit of subtitles becomes apparent once a child ‘can decode basic phonemes’, but ‘the real benefit starts when a child is a sufficiently competent reader that decoding is done subconsciously’ – typically around ages 6–7 in the United Kingdom and the United States (Turn on the Subtitles, n.d.‐a). From that time onwards, same‐language subtitles will allow the child to read ‘a staggering number of words’, equivalent, according to the campaign, to the number of words ‘in all the *Harry Potter* books, all of the *Lord of the Rings*, all of the *Chronicles of Narnia* and everything Roald Dahl wrote, combined’ (Turn on the Subtitles, n.d.‐b; see also Alt, 2024). Indeed, research by Ofcom (2024) suggests that children aged 4–11 years old watch about 5 hr of broadcast television and over 5 hr of paid‐for on‐demand content per week. The campaigns estimate that the average Year 5 child would read the equivalent of 120 pages per day simply by spending 2 hr each day watching television or streaming services with subtitles (Wexler, 2021).

These campaigns have attracted considerable attention and support from celebrities, journalists, media stakeholders and governments (Alt, 2024; BBC Newsround, 2021; Cohen, 2023; Hansard, 2020; Jack Black, 2024; Wexler, 2020, 2021; World Literacy Foundation, 2023; see full list at PlanetRead, n.d.; Turn on the Subtitles, n.d.‐c). The notion that such a simple act – turning on same‐language subtitles – could yield such transformative outcomes has proven irresistible. The campaign has been called ‘the world's largest literacy project’ and compared to ‘sneaking vegetables into dinner, the children don't notice, but you know you're doing them the world of good’ (Cohen, 2023). However, despite claims of strong supporting evidence, few studies have directly tested whether same‐language subtitles actually help children become better readers. Most of the existing research on how readers engage with subtitles has focused on adult readers (d'Ydewalle et al., 1991; Kothari et al., 2004; Kruger & Steyn, 2014; Perego et al., 2010), second‐language learners (Hsieh, 2020; Kothari et al., 2002; Montero Perez et al., 2013; Vanderplank, 2016) and hard‐of‐hearing populations (Koskinen et al., 1986; Yoon & Kim, 2011; see Gernsbacher, 2015 for a review). By contrast, research on children learning to read in their first language remains limited and has serious flaws, or yields findings that contradict the claims made by the campaigns.

One important question is what aspect of reading might be improved by subtitle viewing. Recent research suggests that same‐language subtitles are unlikely to benefit children in the earliest stages of reading acquisition. Lopukhina et al. (2025) conducted a cross‐sectional study with English‐speaking primary school children in Years 1 through 6 (age 5–11) that investigated where children look when watching clips from children's movies with subtitles. Using high‐resolution eye‐tracking, researchers found that children with poor reading fluency tended to ignore subtitles. It was only when children had achieved a reasonable degree of reading fluency, typically in Years 2 or 3 following a few years of reading instruction, that they showed engagement with the subtitles. These results align with earlier findings on pre‐readers who rarely focus on printed text in picture books when being read to and instead look at the illustrations (Evans & Saint‐Aubin, 2005; Justice et al., 2005). The findings of Lopukhina et al. (2025) are important because they constrain when in a pupil's reading journey any possible influence of subtitles on reading might be observed.

There are a small number of intervention studies that included children who should have had basic decoding skills and tested for an influence of same‐language subtitles exposure on measures such as reading fluency, vocabulary and comprehension (see Table 1). However, these studies are characterized by significant methodological shortcomings and also produced inconsistent results. One oft‐quoted study conducted in India tested a large sample of adults and children and reported superior reading across a number of measures amongst participants who reported that they had regularly watched a weekly subtitled programme featuring Bollywood songs, compared to those who rarely or never watched it (Kothari & Bandyopadhyay, 2014). However, the assignment to subtitle versus control groups in this study was not randomized; instead, participants were considered as part of the subtitle or no‐subtitle conditions based on retrospective self‐report (i.e. whether they reported watching the subtitled programme regularly or rarely/never in the past years). In a design like this, it's impossible to attribute any differences between groups to the experience with subtitles, as they could have differed in a variety of ways such as whether participants had easy access to a television (a potential indicator of socio‐economic status). Two laboratory‐based intervention studies that had greater control showed that just a few hours of watching subtitled videos led to improvement in word recognition scores and some aspects of comprehension in English‐speaking children between the ages of 7 and 10 (Linebarger, 2001; Linebarger et al., 2010). However, the findings across these studies were not consistent, and some effects were restricted to particular groups (see Table 1). These findings should be interpreted with caution, as the studies were likely underpowered due to complex interaction analyses in the presence of relatively small sample sizes (Brysbaert, 2019). Further, the different ways of selecting data for analysis in these studies (for example, on the basis of gender) in the absence of preregistration are now known to increase the risk of Type I error (false positives; Nosek et al., 2018).

**TABLE 1:** Summary of major intervention studies on the influence of same‐language subtitles on reading skills in children.

Study	Sample size	Age (mean and/or range in years)	Language	Number of sessions	Subtitle viewing time	Outcomes
Linebarger (2001)	76 4 groups	Means = 8.40 (girls), 8.46 (boys) Range = 7–10 years	English	5	20–30 min	**Positive effect** of subtitles for *target word recognition* and *comprehension* (critical story elements and for boys) **No effect** of subtitles for *oral reading rate* and *spontaneous use of target words* **Negative effect** of subtitles for *comprehension* (character descriptions, main idea, incidental story elements and for girls)
Linebarger et al. (2010)	70 2 groups	Means = 7.5 (girls), 7.8 (boys)	English	6	3 hr	**Positive effect** of subtitles for *high‐frequency (Dolch) words reading*, *nonsense word fluency, comprehension* (target word and inferential), *target word recognition* (for at‐risk children only) **No effect** of subtitles for *oral reading fluency* and *decontextualized target word reading*
Parkhill et al. (2011)	323 3 groups	Range = 11–13 (School years 7–8)	English	30	10–15 hr	**Positive effect** of testing session: experimental and control groups improved their *vocabulary* and *reading comprehension* skills at the post‐test compared to the pretest **No effect** of the intervention type (subtitles)
Kothari and Bandyopadhyay (2014)	2838 2 groups (assigned retrospectively)	Range = 6–14	Hindi	Unknown. The maximum possible number of sessions is 247	Unknown. The maximum possible number of hours is 247	**Positive effect** of subtitles for all 12 measures of *reading*, *writing* and *self‐perception of common functional abilities related to literacy*

*Note*: We report mean age and age ranges based on the information available in the included papers.

There are also a few intervention studies that tested the influence of more deliberate encounters with subtitles on children's reading fluency, vocabulary and comprehension. One set of studies showed that daily explicit interactions with subtitled videos in the classroom over a 6‐week period led to improvements in vocabulary and comprehension among children aged 8 to 15 (Davey & Parkhill, 2014; Parkhill et al., 2011; Parkhill & Davey, 2014). These explicit interactions involved stopping the video and asking children to write down the last subtitle they saw, or to look up a subtitled word in the dictionary. Explicit encounters of this nature with printed words are likely to have different consequences for learning than the incidental encounters that arise when watching subtitled television. Even so, the study by Davey and Parkhill (2014) did not include a control group, making it difficult to determine whether the observed improvement was due to the subtitle intervention or general educational progress. In a study that *did* include a control group (Parkhill et al., 2011), there was no evidence that subtitles improved literacy skills over and above the gains achieved through the school reading curriculum (see Table 1). Other studies testing whether showing only specific words on screen (as opposed to full subtitling) also showed mixed results. Linebarger et al. (2004) reported that 6–7‐year‐old children who watched the animated educational television programme *Between the Lions*, designed to promote reading, outperformed their peers who did not watch the programme in word recognition and early literacy skills. In contrast, Linebarger et al. (2013) found no overall benefit of onscreen print for children aged 4–8 who watched educational videos with or without onscreen words. Taken together, these studies do not offer consistent evidence that deliberate interaction with subtitles or onscreen print improves children's reading.

To summarize, although widely promoted, the claim that same‐language subtitles help children learn to read is not well supported. Much of the existing research has significant methodological limitations, such as flaws in experimental design (e.g. Davey & Parkhill, 2014; Kothari & Bandyopadhyay, 2014) or small sample sizes and low statistical power (e.g. Linebarger, 2001; Linebarger et al., 2010). Moreover, while the campaigns advocate for the benefits of subtitles in naturalistic viewing contexts, most interventions have been conducted in classroom environments (e.g. Linebarger, 2001; Linebarger et al., 2010) and involved explicit instructions to engage with the subtitles (e.g. Parkhill et al., 2011; Parkhill & Davey, 2014). What is needed is a properly‐powered intervention study that tests the effects of subtitle exposure in naturalistic settings, using random assignment to groups and including children who are fluent enough to benefit from same‐language subtitles.

We therefore conducted a preregistered randomized controlled trial testing the causal hypothesis that extended exposure to same‐language subtitles improves children's reading fluency. We focused on reading fluency because of its strong association with reading comprehension (Crawford et al., 2025; Hilton et al., 2024). Influential science journalism has also promoted the view that while subtitles may not help the reading of children who have not learned to decode, they can help to build reading fluency (Wexler, 2020, 2021). Our participants were English‐speaking children who attended Years 2 and 3 of primary school. We chose these age groups because our previous study had confirmed that they have sufficient reading ability to at least look at subtitles (Lopukhina et al., 2025), and because large‐scale data from British school children show an acceleration of reading fluency during these school years (Hilton et al., 2024).

Participants were randomized to experimental and control groups for a 6‐week home‐based intervention. During the intervention, the experimental group watched TV and streaming content with English subtitles, while the control group continued watching without subtitles, as usual. We monitored children's viewing hours and engagement with the study throughout the entire intervention period. We chose a home‐based intervention (rather than a laboratory or classroom intervention) in order to mirror the conditions that children would experience should subtitles be turned on by default for children's programming (as advocated by campaign groups). The dependent measure of reading fluency was operationalized in two ways: through standard tests of reading fluency and through engagement with subtitles while watching short animations, as measured with eye‐tracking. This second set of dependent measures was investigated because our previous work showed that children with higher reading fluency showed greater engagement with subtitles (Lopukhina et al., 2025), and we thought that eye‐tracking measures may capture more subtle improvements in reading fluency than standard assessments. We hypothesized that both groups would show improvement over the 6‐week period due to regular reading activities in the school curriculum. Our main question was whether the experimental group would show greater improvement as a result of their exposure to subtitles.

## METHOD

### Design

The intervention study used a between‐participant design with two groups: intervention with subtitles (experimental group) versus intervention without subtitles (control group). The assignment of participants to groups was randomized using stratified randomization (Torgerson & Torgerson, 2013) with two strata – school year and term of participation. We measured participants' reading skills and their engagement with subtitles before (pretest) and after (post‐test) the home‐based intervention. Our analyses tested whether participants' reading skills and engagement with subtitles improved between pretest and post‐test, and whether this improvement was modulated by the intervention group.

### Participants

Following the preregistered sample size, we recruited 132 children (65 girls and 67 boys) who attended Years 2 and 3 from 36 state‐funded primary schools in England. All children were native English speakers with normal or corrected‐to‐normal vision and hearing, had no special educational needs and watched at least 5 hr of television or streaming services per week without subtitles. Children with special educational needs and those with English as an additional language were excluded because we considered that we would not have sufficient power to contrast children in these groups with typically developing children with English as their main language, and we wanted to reduce heterogeneity in reading trajectories. In our sample, 55 children were tested in England's summer term running from April through July 2024, and 77 children were tested in the autumn term running from September through December 2024. Five children did not complete the intervention and had to be excluded from the study. In total, 127 children were included in the analysis: 64 children in the experimental group (34 girls and 30 boys; mean age = 6.88, range 6–8; 30 in Year 2 and 34 in Year 3) and 63 in the control group (28 girls and 35 boys; mean age = 6.92, range 6–8; 31 in Year 2 and 32 in Year 3).

The study was approved by Royal Holloway's Research Ethics Committee (Reference No: 3484). Children participated based on parental opt‐in written consent, and they were asked on multiple occasions throughout the testing sessions to provide positive verbal assent. Families received monetary compensation for their involvement and children received a gift.

### Materials and procedure

The study included a pretest session, an in‐home intervention and a post‐test session. At the pretest and post‐test, the children were tested individually in schools in an unused classroom (*N* = 87), in a laboratory at the University of Nottingham (*N* = 34), or in a laboratory at Royal Holloway, University of London (*N* = 6). During the *pretest session* (Time 1), all children watched two videos with subtitles and completed measures of reading fluency, reading comprehension and vocabulary. We also had a test of reading habits and attitudes and a measure of print exposure; however, we dropped these tests where the testing session would have exceeded 45–60 min or where children were fatigued. During the *intervention*, children watched television and/or streaming services at home either with subtitles (experimental group) or without subtitles (control group). During the *post‐test session* (Time 2), children watched two different videos with subtitles and completed measures of reading fluency. The post‐test session lasted approximately 20–30 min. Below, we describe the measures and the intervention in more detail (see also Figure 1).

**FIGURE 1:**
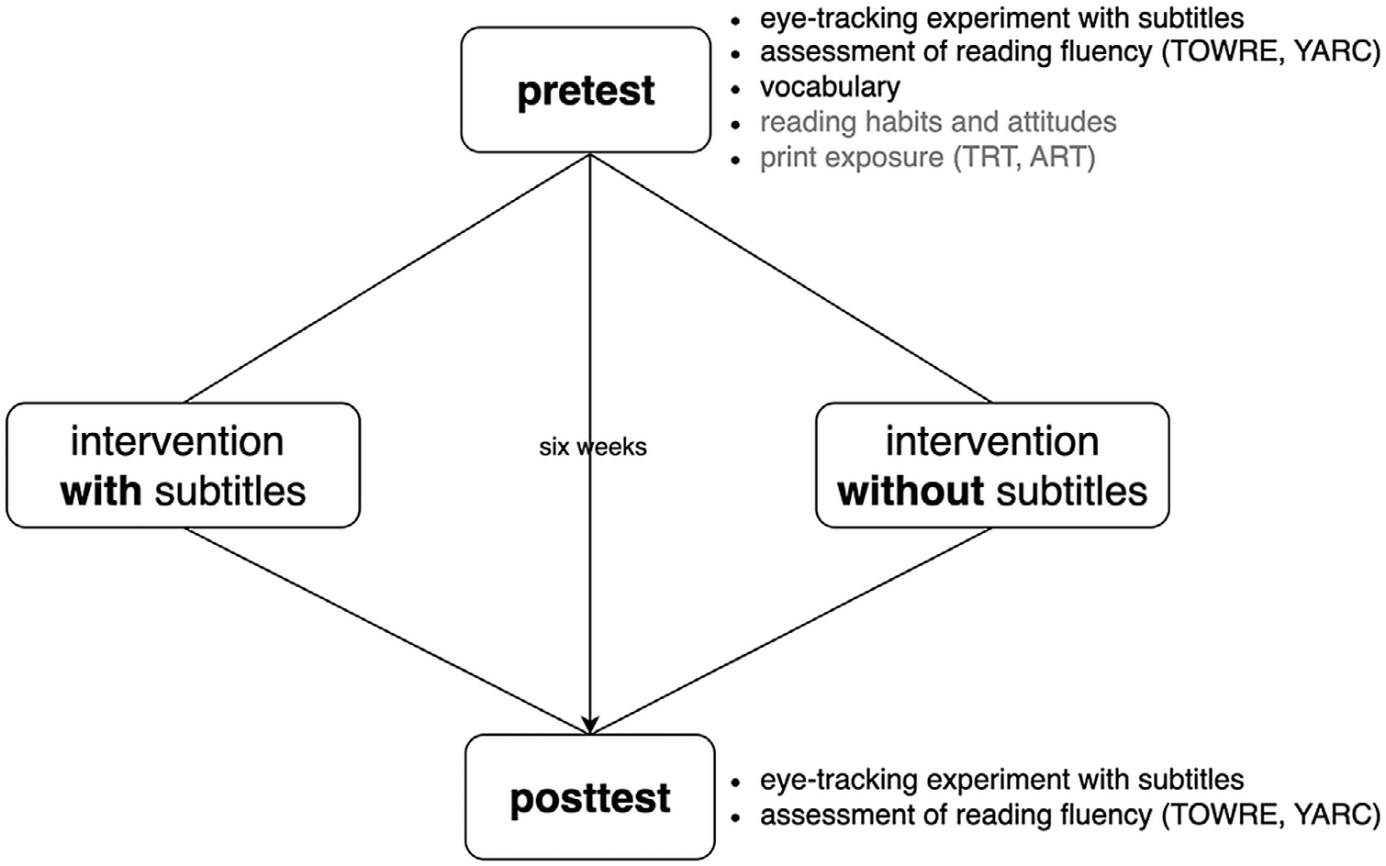
Overview of the experimental procedure, including the order of tasks in the pretest and post‐test sessions. Tasks not completed by all participants are shown in grey.

#### Videos with subtitles (Time 1 and Time 2)

Engagement with subtitles was measured in an eye‐tracking experiment with four short video clips from animated films for children. These video clips were chosen to have adequate language content, to be engaging and to be unfamiliar to children. They were also used in Lopukhina et al. (2025) with primary school children. The videos were grouped into pairs (*Taking Flight*, 5 min 5 s, 64 subtitles + *Rescuers Down Under*, 4 min 21 s, 99 subtitles; and *The Road to El Dorado*, 4 min 43 s, 86 subtitles + *The Wild*, 4 min 13 s, 116 subtitles), so that each participant saw two clips at the pretest and the other two at the post‐test. The assignment of video pairs to the pretest or post‐test was counterbalanced across participants, and the order of the videos within each pair was randomized for each participant. Each video was followed by four comprehension questions addressing one of the main characters, a critical element of the story, the main idea of the story and some incidental content of the story. The questions were used to ensure that children watched the videos for comprehension.

#### Assessments of reading fluency (Time 1 and Time 2)

Reading fluency was measured by two tests – the Test of Word Reading Efficiency – Second Edition (TOWRE; Torgesen et al., 2012) and the York Assessment of Reading for Comprehension (YARC; Stothard et al., 2010). TOWRE is a test that measures how many words and nonwords of increasing length and complexity children can read aloud accurately in 45 s. Raw scores on these two subtests of the test were added together to provide a single measure of word and nonword reading fluency. In YARC, we measured the time required to read aloud a passage at Level 2 (for children in Year 2) or Level 3 (for children in Year 3). The passage was followed by eight questions that assessed literal and inferential understanding, as well as vocabulary knowledge. We administered the comprehension questions at both pre‐ and post‐test, but, as preregistered, we only used the measures from Time 1 to assess whether the two groups of participants were matched (see Table 2). At Time 1 and Time 2, alternate forms of TOWRE and YARC were used (e.g. Form A at the pretest and Form B at the post‐test, or vice versa). Three children in Year 2 inadvertently received passages at Level 3 at both the pre‐ and post‐test.

**TABLE 2:** Means and standard deviations (SDs) of behavioural tests scores at pretest (Time 1).

	Experimental group	Control group
TOWRE	77 (23)	82 (24)
YARC	116 (85)	109 (80)
YARC questions	4 (2)	4 (2)
BPVS	107 (17)	106 (18)
PIRLS	63 (8)	64 (7)
TRT	0.48 (0.1)	0.48 (0.1)
ART	0.47 (0.1)	0.47 (0.1)

*Note*: TOWRE refers to the total number of words and nonwords read in 90 s (45 + 45). YARC refers to the time in seconds required to read a passage of text aloud. YARC questions refer to the number of questions answered correctly (maximum = 8). BPVS refers to raw scores in the receptive vocabulary test. PIRLS is a questionnaire assessing reading habits and attitudes. TRT and ART are tests of print exposure.

#### Vocabulary (Time 1)

Receptive vocabulary was assessed using the British Picture Vocabulary Scales III (BPVS; Dunn et al., 2009). In this test, children were required to identify the meaning of a spoken word from four pictures.

#### Reading habits and attitudes (Time 1)

Reading habits and reading attitudes were assessed using the Progress in International Reading Literacy Study Student questionnaire (PIRLS; questions R1, R4–R8; Reynolds & Martin, 2023). In this questionnaire, on a 4‐point scale, children rated 20 statements grouped under six questions. The first 16 statements probed children's reading lessons, their opinions about reading and their self‐estimation of reading ability. In the other four statements, children were asked to estimate their reading frequency. The experimenter read the questions and statements aloud, and children marked their answers by ticking the appropriate boxes. Each response was scored from 1 (*disagree a lot or read rarely*) to 4 (*agree a lot or read often*), with a maximum total score of 80 points for the test. In total, 103 children completed the questionnaire (53 in the experimental group and 50 in the control group).

#### Print exposure (Time 1)

A title recognition test (TRT) and an author recognition test (ART) were developed to assess print exposure. To construct these tests, we compiled a list of 30 book titles and a list of 30 authors popular among 7–9‐year‐old children using the CYP‐LEX corpus (Korochkina et al., 2024), excluding titles that shared names with popular movies. For each test, an additional 15 foil items were created, resulting in final lists of 45 items each (30 targets and 15 foils). Children were instructed to read the titles or names and tick any they thought were real book titles or the authors of children's books. For some Year 2 children, the titles and author names were read aloud by the experimenter. Scoring was based on the number of targets correctly identified plus the number of foils correctly rejected, divided by the total number of items. In total, 116 participants completed the TRT (58 in each group), and 97 participants completed the ART (51 in the experimental group and 46 in the control group).

#### Intervention

The intervention was home‐based and lasted about 6 weeks (*M* = 41 day; *SD* = 2 days; range 35–44 days). We chose a naturalistic intervention instead of a laboratory study because we aimed to test the claim of the *Turn on the Subtitles* campaign that simply turning on same‐language subtitles will improve children's reading (Turn on the Subtitles, n.d.‐b). Specifically, we wanted to capture typical home viewing conditions, including natural variation in devices, television programmes and engagement (e.g. whether children watched attentively or were distracted or multitasking), rather than a tightly controlled viewing environment.

Prior to the intervention, families received detailed written instructions covering subtitle activation (experimental group), viewing guidance, how feedback would be collected and contact information. Further, to ensure that all participants watched at least some shows with high‐quality subtitles and appropriate language content, we provided the families with a list of suggested shows and series on the children's BBC channel (CBBC) and Netflix. We asked the children to watch at least five episodes from this list per week in addition to (or instead of) their usual television viewing routine. Parents in the experimental group were asked to ensure subtitles were enabled whenever the child watched television or streaming content, and parents in the control group were asked to ensure subtitles were off. Full written instructions for both groups are available on the Open Science Framework. Families were offered support at each stage of the intervention by email and/or phone as needed.

Parents were prompted each weekday by mobile messaging to respond to three questions (1) whether their child watched television/streaming services that day; (2) for how long; (3) whether the subtitles were on/off. On Sunday, we sent the families a survey with questions about television viewing time during the whole week and about the content of the suggested shows or series that the child watched. The purpose of these different forms of communication with parents was both to estimate how much television their children were watching and to ensure that they remained engaged with the intervention across the full 6‐week period. In total, children in the experimental group watched on average 66 hr of television and streaming services with subtitles (*SD* = 29 hr; range = 18–177 hr). Children in the control group watched on average 64 hr of television without subtitles (*SD* = 29 hr; range = 21–197 hr). Based on the background questionnaire, most children watched on a television (95%), with tablets as the secondary device (59%) and smartphones as the third most common device (13%).

A series of simple linear regressions were run to compare performance across seven measures between the experimental and control groups at the pretest. None of the analyses revealed significant differences between the groups (*p*'s > 0.22).

### Apparatus

During the eye‐tracking experiment, children's eye movements were recorded by an EyeLink Portable Duo eye‐tracker (SR Research Ltd) with a sampling rate of 1000 Hz. Stimuli were displayed on an MSi Katana GF76 11UD laptop with a screen resolution of 1920 × 1080 pixels and a 144‐Hz refresh rate. The video resolution was the same as the screen resolution, with subtitles embedded directly in the video. The appearance of each subtitle was synchronized with the audio. The presentation speed of the subtitles was on average 14 characters per second (*SD* = 6.23; range 1–39). The subtitles were displayed in white 28‐point Courier New font against a black background, 82 mm below the screen centre. Each letter of the subtitles subtended ~0.4° of visual angle, calculated based on screen dimensions (384 × 217 mm) and eye‐to‐screen distance of 670 mm. The presentation of subtitles broadly followed the BBC Subtitle Guidelines (BBC, 2025). Viewing was binocular, but only the right eye was tracked. A chin rest was used to minimize head movements.

The experiment started with a 9‐point calibration and validation procedure, repeated as necessary until a mean accuracy of 0.5° of visual angle or better was achieved. The presentation of each video started with a drift correction at the centre of the screen. Participants were instructed to watch videos and to answer comprehension questions following each video.

### Analysis

Our preregistered analyses examined children's eye‐movement behaviour as a function of intervention group, testing session and the interaction between these variables. These analyses then assessed whether the effect of the subtitle intervention transferred to independent measures of reading fluency. Finally, we conducted an exploratory analysis to investigate whether children who spent more time watching television with subtitles showed greater improvement in reading fluency and increased engagement with subtitles.

We analysed children's global eye‐movement behaviour while watching videos with subtitles, as well as their eye movements at the level of individual words in subtitles. For the global analysis, we divided the screen into the subtitle region and the main scene region above the subtitles (see Figure S1). We report five preregistered eye‐movement measures:

1. *number of fixations in the subtitle region*. The proportion of fixations in the subtitle region at the level of individual subtitles was calculated and then these subtitle‐level proportions were averaged across all subtitles in each video for each participant, yielding a per‐video measure of fixations in the subtitle region.
2. *fixation durations in the subtitle region*. The proportion of all fixation durations in the subtitle region at the level of individual subtitles was calculated and then these subtitle‐level proportions were averaged across all subtitles in each video for each participant to obtain per‐video measures.
3. *crossovers*. For each subtitle, saccades that moved between the subtitle region and the main scene in either direction were identified and coded as 1; within‐region saccades were coded as 0. For each participant and video, the mean crossover value across all saccades was computed, yielding a per‐video proportion of crossovers.
4. *skipped subtitles*. For each subtitle, skipping was coded as 1 when no fixations landed within the subtitle region, and 0 otherwise. For each participant and video, the mean skipping value across all subtitles was computed, yielding a per‐video proportion of skipped subtitles.
5. *skipped words*. For the subtitles that were not fully skipped, each word was coded as 1 when it was skipped, and 0 otherwise. For each participant and video, the mean skipping value across all words was computed, yielding a per‐video proportion of skipped words.

In line with Lopukhina et al. (2025), no data cleaning was applied prior to analysis because the paradigm was very different from typical experiments on reading that use eye tracking. The stimuli involved video clips with dynamic text presentation and simultaneous audio presentation. Thus, a conservative approach was used, given evidence that the choice of data cleaning procedure can have an impact on the observed effects (Eskenazi, 2023).

Reading fluency was assessed using *TOWRE* (total number of words and nonwords read correctly) and *YARC* (time in seconds to read a passage aloud). The TOWRE score also incorporates reading accuracy, while our YARC measure focused solely on reading speed. Scoring was conducted by the raters who were blind to experimental conditions.

Data were analysed using linear mixed‐effects models (Baayen et al., 2008). Analyses were conducted with R (R Core Team, 2025). Models were estimated with the ‘lme4’ package (Bates et al., 2015), and tables showing model outcomes were created with the ‘sjPlot’ package (Lüdecke, 2017). For the fixed effects structure, intervention group and testing session were both coded with sum contrasts (1 referred to the experimental group and to the post‐test session; −1 to the control group and to the pretest session; Schad et al., 2020). Sum contrast coding centres categorical predictors, so coefficients reflect deviations from the grand mean, facilitating the interpretation of effects in models with interactions. The total number of hours of TV viewing was treated as a continuous variable and centred on the grand mean. All models included random intercepts for participants. The optimal models for the eye‐tracking measures also included random intercepts for videos, as well as by‐participant random slopes for session. We started with the maximal model and optimized its random structure so that it was best supported by both the experimental design and the data (Bates et al., 2018).

We report unstandardized effect sizes in the form of *β* coefficients, along with data means and standard deviations. The *β* value represents the fixed‐effect coefficient, that is, the estimated difference between conditions after accounting for random effects. For example, for the fixed effect of session (coded as 1 post‐test, −1 pretest), a *β* of 1.47 would indicate that participants read aloud 2.94 more words and nonwords in TOWRE at post‐test compared to pretest.

## RESULTS

One participant did not complete the eye tracking experiment at post‐test, and their eye‐movement data were excluded from the analysis. During the debriefing, two children indicated that the characters of *The Road to El Dorado* seemed familiar, but they did not remember any details of the film. We decided that this level of familiarity was not sufficient to exclude them from the study.

### Engagement with subtitles

The first series of analyses tested whether participants looked at the subtitle region more and for a longer duration at post‐test relative to pretest, and whether this effect was greater for participants in the experimental group than in the control group.

#### Fixations (number)

Statistical analysis showed no main effect of session (*β* = 0.00, *SE* = 0.00, *t* = 0.85, *p* = .397) or group (*β* = −0.01, *SE* = 0.01, *t* = −0.40, *p* = .691), or a group‐by‐session interaction (*β* = 0.00, *SE* = 0.00, *t* = 0.42, *p* = .672) for the number of fixations in the subtitle region, indicating no change from pretest to post‐test in either group of children (see Table 3 for a summary of the means). All model outputs are presented in Table S2.

**TABLE 3:** Means and standard deviations (in parentheses) for eye‐movement (reported as proportions) and reading fluency measures in the two groups at pretest and post‐test.

		Proportion of fixations	Proportion of fixation duration	Proportion of crossovers	Proportion of skipped subtitles	Proportion of skipped words	TOWRE (number correct)	YARC (s)
Experimental group	Pretest	0.373 (0.172)	0.360 (0.173)	0.210 (0.069)	0.326 (0.23)	0.615 (0.108)	77 (23)	116 (85)
	Post‐test	0.384 (0.154)	0.367 (0.152)	0.233 (0.078)	0.287 (0.23)	0.606 (0.089)	82 (22)	105 (66)
Control group	Pretest	0.387 (0.185)	0.377 (0.186)	0.221 (0.089)	0.306 (0.273)	0.579 (0.114)	82 (24)	109 (80)
	Post‐test	0.391 (0.168)	0.376 (0.167)	0.233 (0.086)	0.287 (0.244)	0.590 (0.107)	84 (23)	100 (78)

*Note*: Higher TOWRE values indicate superior reading fluency while higher YARC values indicate poorer reading fluency.

#### Fixations (duration)

Similarly, for fixation durations in the subtitle region, no main effect of session (*β* = 0.00, *SE* = 0.00, *t* = 0.41, *p* = .680) or group (*β* = −0.01, *SE* = 0.01, *t* = −0.49, *p* = .624), or a group‐by‐session interaction was observed (*β* = 0.00, *SE* = 0.00, *t* = 0.48, *p* = .633). These results also indicate that there was no change in fixation durations on subtitles from pretest to post‐test in either group of children.

#### Crossovers

Children made more crossovers between the main scene and the subtitle regions at the post‐test compared to the pre‐test (*β* = 0.01, *SE* = 0.00, *t* = 3.68, *p* < .001). There was no main effect of group (*β* = −0.00, *SE* = 0.01, *t* = −0.42, *p* = .674) or group‐by‐session interaction (*β* = 0.00, *SE* = 0.00, *t* = 1.20, *p* = .233), indicating that the effect was comparable for both groups of children (see Figure 2a).

**FIGURE 2:**
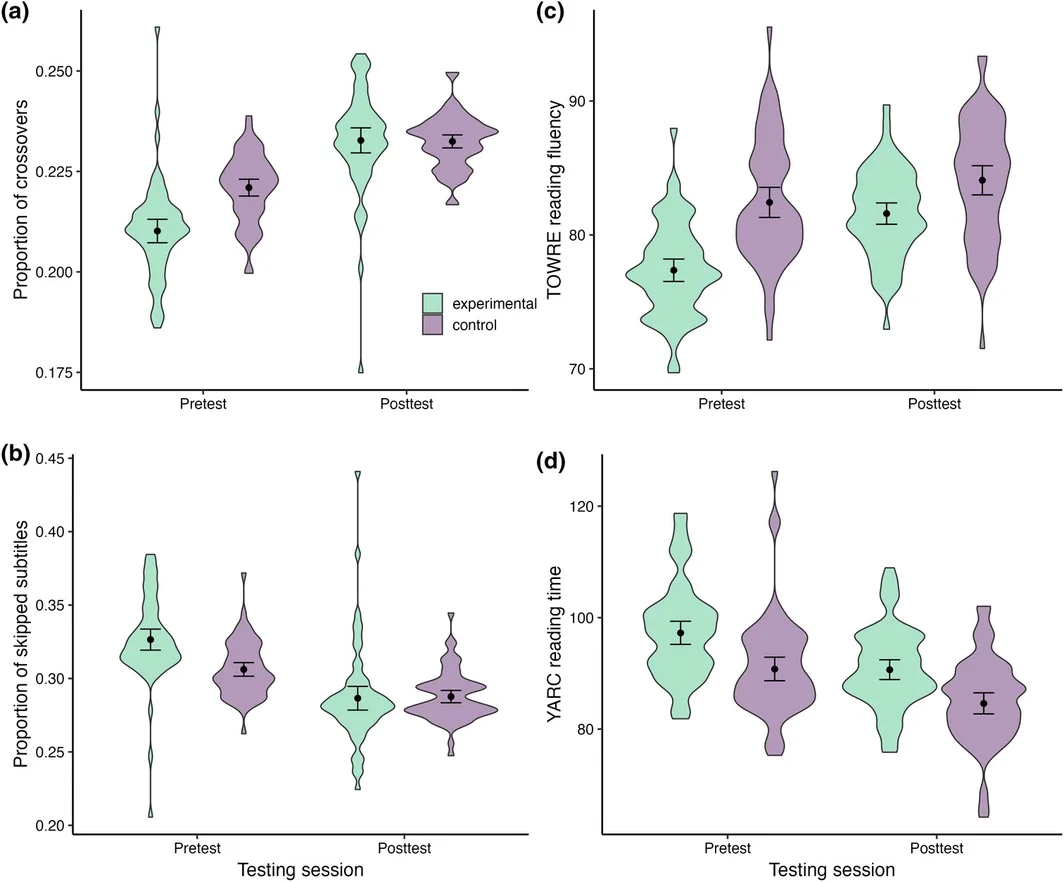
Estimates of eye movement and reading fluency measures at pretest and post‐test. Panels present only those measures for which significant effects were found; non‐significant outcomes are reported in the text. Panel (a): The effect of testing session by group (experimental – green; control – purple) on the proportion of crossovers (saccades between subtitle and main scene regions, and vice versa). Panel (b): The effect of testing session by group on the proportion of skipped subtitles. Panel (c): The effect of testing session by group on the number of words and nonwords read aloud in TOWRE. Panel (d): The effect of testing session by group on YARC reading time in seconds. Error bars represent 95% CIs.

#### Skipped subtitles

Children skipped fewer subtitles at the post‐test compared to the pretest (*β* = −0.01, *SE* = 0.01, *t* = −2.39, *p* = .017). We found no main effect of group (*β* = 0.00, *SE* = 0.02, *t* = 0.25, *p* = .804) or group‐by‐session interaction (*β* = −0.01, *SE* = 0.01, *t* = −0.87, *p* = .382), indicating that the effect was comparable for both groups (see Figure 2b).

#### Skipped words

Focusing only on subtitles that were not skipped, no effect of session (*β* = 0.00, *SE* = 0.00, *t* = 0.25, *p* = .805), group (*β* = 0.01, *SE* = 0.01, *t* = 1.54, *p* = .124), or group‐by‐session interaction (*β* = −0.00, *SE* = 0.00, *t* = −1.88, *p* = .060) on the proportion of skipped words was observed, indicating that there was no change from pretest to post‐test in either group of children.

### Reading fluency

The second series of analyses tested whether reading fluency improved from pretest to post‐test, and whether this improvement was greater for children in the experimental group relative to the control group.

#### TOWRE

Children read aloud more items in TOWRE at the post‐test compared to the pretest (*β* = 1.47, *SE* = 0.34, *t* = 4.30, *p* < .001). There was no main effect of group (*β* = −1.89, *SE* = 1.96, *t* = −0.97, *p* = .335) or group‐by‐session interaction (*β* = 0.65, *SE* = 0.34, *t* = 1.89, *p* = .060), indicating that there is insufficient evidence to reject the null hypothesis of no effect (see Figure 2c). All model outputs are presented in Table S3.

#### YARC

Children's passage reading fluency improved from pretest to post‐test (*β* = −0.04, *SE* = 0.01, *t* = −4.60, *p* < .001). However, no main effect of group (*β* = 0.03, *SE* = 0.05, *t* = 0.73, *p* = .468) or group‐by‐session interaction was observed (*β* = 0.00, *SE* = 0.01, *t* = 0.00, *p* = .999), indicating that the improvement was comparable for both groups (see Figure 2d).

### Exploratory analyses

#### Amount of intervention

This set of analyses examined whether the total number of hours children spent watching television and streaming services during the intervention was associated with greater engagement with subtitles and higher reading fluency, specifically in the experimental group but not in the control group. Across all statistical models, no main effect of the number of hours of intervention, nor any significant interactions between hours of intervention and other predictors were observed. These findings suggest that increased exposure to television with subtitles did not influence children's engagement with subtitles or their reading fluency. All model outputs are presented in Tables S4 and S5.

## DISCUSSION

The aim of this study was to investigate the claim that turning on same‐language subtitles helps children become better readers. Specifically, we tested the causal hypothesis that exposure to subtitles improves children's reading fluency. We conducted an intervention study with 127 children in Years 2 and 3, who should have had sufficient reading skills to engage with subtitles (Lopukhina et al., 2025) and were actively developing their reading fluency (Hilton et al., 2024). The children's reading fluency was measured using two standard assessments and by recording their eye movements while they were watching videos with subtitles, both before and after a 6‐week home‐based intervention. During the intervention, the children were randomly assigned to either an experimental group who watched television and streaming content with English subtitles on, or a control group who watched everything without subtitles. To the best of our knowledge, this is the first preregistered, adequately powered randomized control trial exploring whether watching television with same‐language subtitles can help children improve their reading fluency.

Our main finding was that both groups of children improved their reading fluency between the pretest and the post‐test. The effect of the testing session was observed in both of our behavioural measures of reading fluency (TOWRE and YARC) and in two of our eye‐movement measures (the proportion of crossovers and the proportion of skipped subtitles). Crucially, there was no evidence that 6 weeks of exposure to subtitles drove an improvement in reading fluency, or that children who watched more subtitled television showed any additional benefit. Thus, we conclude that while children's typical reading experiences at this age have a positive impact on reading fluency that is detectable over just 6 weeks, there is no additional benefit of subtitles exposure in naturalistic settings.

One question is whether more extended exposure to subtitles would result in an influence on reading fluency. In our study, participants underwent a 6‐week intervention, and children in the experimental group had, on average, 66 hr of total exposure to subtitles. This intervention ‘dose’ is between 22 and 132 times greater than that in the controlled studies promoted by subtitles campaigns (Linebarger, 2001; Linebarger et al., 2010). It is important to highlight that with this amount of exposure, we were only able to detect the effect of the testing session itself, which we interpret as the benefit of reading instruction at school, but it was insufficient to produce an effect of subtitles. If there is a beneficial effect of subtitles on reading fluency, it must therefore be small – much smaller than the effect of school education. Future studies should keep in mind the methodological implications raised by our results: any possible effect of subtitles on reading skills is likely to be considerably weaker than the robust effects of school‐based reading curricula.

Another question is whether more deliberate interactions with subtitles could influence reading outcomes; for example, if parents or teachers actively direct children to engage with subtitles. However, part of the appeal of the *Turn on the Subtitles* campaign is that it promises substantial returns for very low effort. Once subtitles are on screen, they cannot be ignored and automatically ‘turn screen time into reading time’ (Switch On Read On, n.d.; Turn on the Subtitles, n.d.‐d; Wexler, 2021), allowing children to ‘learn to read without even realising it’ (Jack Black, 2024). Our intervention was therefore designed to recreate typical conditions under which children encounter subtitles at home. We acknowledge that a key limitation of a home‐based, naturalistic intervention is that we were unable (and did not wish) to control whether children consistently attended to subtitles while watching video content. Further, as we did not wish to interfere with children's usual viewing routines, parents were not instructed to prompt children to read the subtitles. However, this limitation also applies to real‐world subtitle campaigns: children may multitask, become distracted or watch while tired, and may not consistently attend to the subtitles. It is possible that other types of subtitle interventions, for example, those that explicitly instruct children to attend to subtitles during viewing and perform subtitle‐related tasks (e.g. Parkhill et al., 2011; Parkhill & Davey, 2014), could yield different effects on reading outcomes. This could be a question for further investigation.

Our study suggests that exposure to same‐language subtitles is unlikely to yield meaningful improvements in children's reading fluency. This raises the question of whether subtitles have potential for supporting other aspects of learning or benefiting groups beyond our sample. It's possible that same‐language subtitles could promote vocabulary learning in children who already read fluently. For example, children who speak English as an additional language (EAL) often develop strong reading fluency but lag behind on measures of reading comprehension due to their English proficiency (Murphy, 2017). EAL children represent about 21% of primary‐school pupils in the United Kingdom (Department for Education, 2025). These children require explicit vocabulary instruction and additional practice, which could potentially, to some extent, be supported by watching television with same‐language subtitles. However, this hypothesis would need to be substantiated with evidence. We also acknowledge that our sample excluded children with special educational needs related to language and/or literacy, and therefore our conclusions should not be generalized to this group without further evidence. It is possible that some children with reading difficulties (e.g. dyslexia) might benefit differently from subtitle exposure. However, we also note that children with reading difficulties often struggle to decode printed words fluently (Snowling et al., 2020), and it is therefore likely that they would struggle to read subtitles given the limited time they remain on screen (Lopukhina et al., 2025). Future work should examine subtitle engagement and outcomes in children with special educational needs separately, possibly with designs that include additional support or instruction to encourage attention to subtitles.

Even if there were a role for same‐language subtitles in promoting vocabulary learning, it's important to consider that the vocabulary found in subtitles of children's television programmes is much less sophisticated than the vocabulary used in children's books. In fact, about 40% of the words used in children's books do not appear in BBC television programmes aimed at children of the same age, suggesting that books provide a unique opportunity to enhance children's vocabulary (Korochkina et al., 2024; see also Dong & Nation, 2025; Nation et al., 2022). These data suggest that the claim of the *Turn on the Subtitles* campaign – that children who watch television with same‐language subtitles would encounter as many words ‘as in all the *Harry Potter* books, all of *The Lord of the Rings*, all of *The Chronicles of Narnia*, and everything Roald Dahl wrote, combined’ (Turn on the Subtitles, n.d.‐b; see also Alt, 2024) – may be correct in terms of quantity but not in terms of the quality of words. Book language plays a pivotal role in developing children's literacy, oral language and social abilities, providing them with tools to communicate about experiences beyond everyday situations (Nation et al., 2022).

Future research investigating whether subtitles influence children's literacy should consider the mechanism through which learning might occur. One possibility is that learning takes place when children match the oral and printed forms of a word. For example, when children see words in subtitles alongside visual information in the video and hear the accompanying audio, this multimodal presentation may strengthen the encoding, storage and retrieval of the word (Linebarger et al., 2010). However, we cannot be certain that children actually look at a particular word in a subtitle when they hear it in the audio. Lopukhina et al. (2025) found that even when primary school children attend to a subtitle, they skip between 50% and 66% of the words in that subtitle. This finding suggests that a substantial proportion of the words in subtitles are not fixated and may not be processed visually. In the present study, the proportion of skipped words in videos among children in Years 2–3 was, on average, 58%–61%, also indicating that many words in subtitles were not fixated. Future studies should explore whether children attempt to fixate on words precisely when those words are pronounced in the audio – a process that might support vocabulary acquisition.

## CONCLUSION

In conclusion, our study shows that watching television or streaming content with same‐language subtitles over a 6‐week period does not yield improvements in the reading fluency of children in Years 2–3 (ages 6–8) beyond the progress achieved through regular reading experiences at school and at home. Overall, despite great enthusiasm around global subtitles campaigns, we do not believe there is compelling evidence to support the claim that same‐language subtitles support children's reading acquisition. Across the available evidence, a consistent pattern suggests that when children are still developing basic decoding skills, they do not attend to subtitles and therefore cannot benefit from them (Lopukhina et al., 2025). Once children reach sufficient reading fluency to attend to subtitles, they are typically already proficient enough that incidental exposure yields little measurable benefit, particularly compared with book reading, which provides richer and more sustained language input. Evidence‐based reading instruction at school and supervised book reading at home are effective ways to develop strong, confident readers (Castles et al., 2018). Policymakers and literacy charities should focus on finding the best ways to implement evidence‐based reading instruction in schools and to support children in reading more at home, rather than promoting same‐language subtitles as a tool for improving reading skills.

## AUTHOR CONTRIBUTIONS

**Anastasiya Lopukhina:** Conceptualization; investigation; formal analysis; data curation; writing – original draft; writing – review and editing; methodology. **Holly Cooper:** Investigation; writing – review and editing. **Cheng‐Yu Hsieh:** Investigation; writing – review and editing. **Walter J. B. van Heuven:** Conceptualization; writing – review and editing; funding acquisition; methodology. **Kathleen Rastle:** Conceptualization; investigation; writing – original draft; writing – review and editing; funding acquisition; methodology.

## CONFLICT OF INTEREST DECLARATION

The authors declare no conflicts of interest.

## Supporting information


**Data S1:** Supporting Information.

## Data Availability

The experiment was preregistered (https://doi.org/10.17605/OSF.IO/F8KAR). Stimulus materials, data, analysis script and Supplemental Materials are available on the Open Science Framework (https://doi.org/10.17605/OSF.IO/XP5AC). Minor deviations are reported in Table S1.

## References

[bjop70066-bib-0001] Alt, C. (2024). Turn on TV subtitles to help children with reading, parents told. *The Times*. https://www.thetimes.com/uk/article/tv‐subtitles‐children‐reading‐skills‐literacy‐j2l735zmj

[bjop70066-bib-0002] Baayen, R. H. , Davidson, D. J. , & Bates, D. M. (2008). Mixed‐effects modeling with crossed random effects for subjects and items. Journal of Memory and Language, 59(4), 390–412. 10.1016/j.jml.2007.12.005

[bjop70066-bib-0003] Bates, D. , Kliegl, R. , Vasishth, S. , & Baayen, H. (2018). Parsimonious mixed models. *arXiv preprint* arXiv:1506.04967.

[bjop70066-bib-0004] Bates, D. , Mächler, M. , Bolker, B. , & Walker, S. (2015). Fitting linear mixed‐effects models using lme4. Journal of Statistical Software, 67, 1–48. 10.18637/jss.v067.i01

[bjop70066-bib-0005] BBC . (2025). BBC subtitle guidelines. https://www.bbc.co.uk/accessibility/forproducts/guides/subtitles/

[bjop70066-bib-0006] BBC Newsround . (2021). Want to be a better reader? Watch the telly with subtitles on! Newsround. https://www.bbc.co.uk/newsround/56211735

[bjop70066-bib-0007] Brysbaert, M. (2019). How many participants do we have to include in properly powered experiments? A tutorial of power analysis with reference tables. Journal of Cognition, 2(1), 1–38. 10.5334/joc.72 PMC6640316 31517234

[bjop70066-bib-0008] Castles, A. , Rastle, K. , & Nation, K. (2018). Ending the Reading wars: Reading acquisition from novice to expert. Psychological Science in the Public Interest, 19(1), 5–51. 10.1177/1529100618772271 29890888

[bjop70066-bib-0009] Cohen, T. F. (2023). Parents: Turn on the TV subtitles! There's good evidence that it will help your child learn to read. Slate. https://slate.com/technology/2023/09/screen‐time‐kids‐benefit‐subtitles‐literacy‐reading.html

[bjop70066-bib-0010] Cole, A. , Brown, A. , Clark, C. , & Picton, I. (2022). Children and young people's reading engagement in 2022. Continuing insight into the impact of the Covid19 pandemic on reading. National Literacy Trust. https://nlt.cdn.ngo/media/documents/Reading_trends_2022_‐_Final.pdf

[bjop70066-bib-0011] Crawford, M. , Raheel, N. , Korochkina, M. , & Rastle, K. (2025). Inadequate foundational decoding skills constrain global literacy goals for pupils in low‐and middle‐income countries. Nature Human Behaviour, 9(1), 74–83. 10.1038/s41562-024-02028-x PMC11774748 39516405

[bjop70066-bib-0012] Davey, R. , & Parkhill, F. (2014). Reading while viewing: The impact of movie subtitles as a strategy to raise achievement in comprehension and vocabulary. Pacific‐Asian Education, 26(2), 21–36.

[bjop70066-bib-0013] Department for Education . (2025). *School pupils and their characteristics: Academic year 2024/25*. Explore education statistics. https://explore‐education‐statistics.service.gov.uk/find‐statistics/school‐pupils‐and‐their‐characteristics/2024‐25

[bjop70066-bib-0014] Dong, Y. , & Nation, K. (2025). Charting the frequency and diversity of emotion words in children's language: Written language matters. First Language, 45(4), 457–475. 10.1177/01427237251339788

[bjop70066-bib-0015] Dunn, L. M. , Dunn, D. M. , Styles, B. , & Sewell, J. (2009). The British Picture Vocabulary Scale (3rd ed.). GL Assessment Limited.

[bjop70066-bib-0016] d'Ydewalle, G. , Praet, C. , Verfaillie, K. , & Rensbergen, J. V. (1991). Watching subtitled television: Automatic reading behavior. Communication Research, 18(5), 650–666. 10.1177/009365091018005005

[bjop70066-bib-0017] Eskenazi, M. A. (2023). Best practices for cleaning eye movement data in reading research. Behavior Research Methods, 56, 1–11. 10.3758/s13428-023-02137-x 37222925

[bjop70066-bib-0018] Evans, M. A. , & Saint‐Aubin, J. (2005). What children are looking at during shared storybook reading: Evidence from eye movement monitoring. Psychological Science, 16(11), 913–920. 10.1111/j.1467-9280.2005.01636.x 16262779

[bjop70066-bib-0019] Gernsbacher, M. A. (2015). Video captions benefit everyone. Policy Insights From the Behavioral and Brain Sciences, 2(1), 195–202. 10.1177/2372732215602130 PMC5214590 28066803

[bjop70066-bib-0020] Hansard . (2020). Commons chamber. Volume 685: Debated on Wednesday 9 December 2020 [Oral Answers to Questions]. Hansard. https://hansard.parliament.uk/Commons/2020‐12‐09/debates/3f2870d8‐1c32‐49a0‐8658‐6382725fcbe1/CommonsChamber

[bjop70066-bib-0021] Hilton, E. , Fischer, M. , Charman, P. , Plaister, N. , Thomson, D. , & Rastle, K. (2024). Measuring reading fluency during primary education. FFT Education Datalab. https://ffteducationdatalab.org.uk/2024/11/measuring‐reading‐fluency‐during‐primary‐education/

[bjop70066-bib-0022] Hsieh, Y. (2020). Effects of video captioning on EFL vocabulary learning and listening comprehension. Computer Assisted Language Learning, 33(5–6), 567–589. 10.1080/09588221.2019.1577898

[bjop70066-bib-0023] Jack Black . (2024). Here's an awesome tip for helping children with their reading. Did you know… [Video]. TikTok. https://www.tiktok.com/@jackblack/video/7348593054007250218

[bjop70066-bib-0024] Justice, L. M. , Skibbe, L. , Canning, A. , & Lankford, C. (2005). Pre‐schoolers, print and storybooks: An observational study using eye movement analysis. Journal of Research in Reading, 28(3), 229–243. 10.1111/j.1467-9817.2005.00267.x

[bjop70066-bib-0025] Korochkina, M. , Marelli, M. , Brysbaert, M. , & Rastle, K. (2024). The Children and Young People's Books Lexicon (CYP‐LEX): A large‐scale lexical database of books read by children and young people in the United Kingdom. Quarterly Journal of Experimental Psychology, 77(12), 2418–2438. 10.1177/17470218241229694 PMC11607844 38262912

[bjop70066-bib-0026] Koskinen, P. S. , Wilson, R. M. , & Jensema, C. J. (1986). Using closed‐captioned television in the teaching of reading to deaf students. American Annals of the Deaf, 131(1), 43–46. 10.1353/aad.2012.0751 3716959

[bjop70066-bib-0027] Kothari, B. , & Bandyopadhyay, T. (2014). Same language subtitling of Bollywood film songs on TV: Effects on literacy. Information Technologies & International Development, 10(4), 31–47.

[bjop70066-bib-0028] Kothari, B. , Pandey, A. , & Chudgar, A. R. (2004). Reading out of the “idiot box”: Same‐language subtitling on television in India. Information Technologies & International Development, 2(1), 23–44.

[bjop70066-bib-0029] Kothari, B. , Takeda, J. , Joshi, A. , & Pandey, A. (2002). Same language subtitling: A butterfly for literacy? International Journal of Lifelong Education, 21(1), 55–66. 10.1080/02601370110099515

[bjop70066-bib-0030] Kruger, J. , & Steyn, F. (2014). Subtitles and eye tracking: Reading and performance. Reading Research Quarterly, 49(1), 105–120. 10.1002/rrq.59

[bjop70066-bib-0031] Linebarger, D. , Piotrowski, J. T. , & Greenwood, C. R. (2010). On‐screen print: The role of captions as a supplemental literacy tool. Journal of Research in Reading, 33(2), 148–167. 10.1111/j.1467-9817.2009.01407.x

[bjop70066-bib-0032] Linebarger, D. L. (2001). Learning to read from television: The effects of using captions and narration. Journal of Educational Psychology, 93(2), 288–298. 10.1037/0022-0663.93.2.288

[bjop70066-bib-0033] Linebarger, D. L. , Kosanic, A. Z. , Greenwood, C. R. , & Doku, N. S. (2004). Effects of viewing the television program between the lions on the emergent literacy skills of young children. Journal of Educational Psychology, 96(2), 297–308. 10.1037/0022-0663.96.2.297

[bjop70066-bib-0034] Linebarger, D. L. , Moses, A. , Garrity Liebeskind, K. , & McMenamin, K. (2013). Learning vocabulary from television: Does onscreen print have a role? Journal of Educational Psychology, 105(3), 609–621. 10.1037/a0032582

[bjop70066-bib-0035] Lopukhina, A. , van Heuven, W. J. , Crowley, R. , & Rastle, K. (2025). Where do children look when watching videos with same‐language subtitles? Psychological Science, 36(4), 223–236. 10.1177/09567976251325789 PMC13428831 40172579

[bjop70066-bib-0036] Lüdecke, D. (2017). sjPlot: Data visualization for statistics in social science. https://CRAN.R‐project.org/package=sjPlot

[bjop70066-bib-0037] Montero Perez, M. , Van Den Noortgate, W. , & Desmet, P. (2013). Captioned video for L2 listening and vocabulary learning: A meta‐analysis. System, 41(3), 720–739. 10.1016/j.system.2013.07.013

[bjop70066-bib-0038] Mullis, I. V. S. , von Davier, M. , Foy, P. , Fishbein, B. , Reynolds, K. A. , & Wry, E. (2023). PIRLS 2021 international results in Reading. Boston College, TIMSS & PIRLS International Study Center. 10.6017/lse.tpisc.tr2103.kb5342

[bjop70066-bib-0039] Murphy, V. A. (2017). Literacy development in children with English as an additional language (EAL). In J. Enever & E. Lindgren (Eds.), Early language learning: Complexity and mixed methods (pp. 41–61). Multilingual Matters. 10.21832/9781783098323-006

[bjop70066-bib-0040] Nation, K. , Dawson, N. J. , & Hsiao, Y. (2022). Book language and its implications for children's language, literacy, and development. Current Directions in Psychological Science, 31(4), 375–380. 10.1177/09637214221103264

[bjop70066-bib-0041] National Literacy Trust . (2024). Children and young people's reading in 2024. National Literacy Trust. https://literacytrust.org.uk/research‐services/research‐reports/children‐and‐young‐peoples‐reading‐in‐2024/

[bjop70066-bib-0042] Nosek, B. A. , Ebersole, C. R. , DeHaven, A. C. , & Mellor, D. T. (2018). The preregistration revolution. Proceedings of the National Academy of Sciences of the United States of America, 115(11), 2600–2606. 10.1073/pnas.1708274114 PMC5856500 29531091

[bjop70066-bib-0043] Ofcom . (2024). Children and parents: Media use and attitudes report 2024. Explore education statistics. https://www.ofcom.org.uk/siteassets/resources/documents/research‐and‐data/media‐literacy‐research/children/children‐media‐use‐and‐attitudes‐2024/childrens‐media‐literacy‐report‐2024.pdf

[bjop70066-bib-0044] Parkhill, F. , & Davey, R. (2014). ‘I used to read one page in two minutes and now I am reading ten’: Using popular film subtitles to enhance literacy outcomes. Literacy Learning: The Middle Years, 22(2), 28–33.

[bjop70066-bib-0045] Parkhill, F. , Johnson, J. , & Bates, J. (2011). Capturing literacy learners: Evaluating a reading programme using popular novels and films with subtitles. Digital Culture & Education, 3(2), 140–156.

[bjop70066-bib-0046] Perego, E. , Del Missier, F. , Porta, M. , & Mosconi, M. (2010). The cognitive effectiveness of subtitle processing. Media Psychology, 13(3), 243–272. 10.1080/15213269.2010.502873

[bjop70066-bib-0047] PlanetRead . (n.d.). Policy support. PlanetRead. https://www.planetread.org/policysupport

[bjop70066-bib-0048] R Core Team . (2025). R: A language and environment for statistical computing (Version 4.4.3). R Foundation for Statistical Computing. https://www.R‐project.org/

[bjop70066-bib-0049] Reynolds, K. , & Martin, M. O. (2023). Updating the PIRLS 2021 instruments for describing the contexts for student learning. In M. von Davier , I. V. S. Mullis , B. Fishbein , & P. Foy (Eds.), Methods and procedures: PIRLS 2021 technical report (pp. 2.1–2.8). Boston College, TIMSS & PIRLS International Study Center. 10.6017/lse.tpisc.tr2102.kb8382

[bjop70066-bib-0050] Schad, D. J. , Vasishth, S. , Hohenstein, S. , & Kliegl, R. (2020). How to capitalize on a priori contrasts in linear (mixed) models: A tutorial. Journal of Memory and Language, 110, 104038. 10.1016/j.jml.2019.104038

[bjop70066-bib-0051] Snowling, M. J. , Hulme, C. , & Nation, K. (2020). Defining and understanding dyslexia: Past, present and future. Oxford Review of Education, 46(4), 501–513. 10.1080/03054985.2020.1765756 PMC7455053 32939103

[bjop70066-bib-0052] Stothard, S. , Hulme, C. , Clarke, P. J. , Barnby, P. , & Snowling, M. (2010). The York Assessment of Reading for Comprehension (YARC): Passage reading secondary. GL Assessment.

[bjop70066-bib-0053] Switch On Read On . (n.d.). Switbch On | Read On. https://switchonreadon.org/

[bjop70066-bib-0054] Torgerson, C. J. , & Torgerson, D. J. (2013). Randomised trials in education: An introductory handbook. Education Endowment Foundation.

[bjop70066-bib-0055] Torgesen, J. K. , Wagner, R. K. , & Rashotte, C. A. (2012). TOWRE‐2 examiner's manual. Pro‐Ed.

[bjop70066-bib-0056] Turn on the Subtitles . (n.d.‐a). FAQ. https://turnonthesubtitles.org/faq/

[bjop70066-bib-0057] Turn on the Subtitles . (n.d.‐b). Parents. https://turnonthesubtitles.org/parents/

[bjop70066-bib-0058] Turn on the Subtitles . (n.d.‐c). Supporters. https://turnonthesubtitles.org/supporters/

[bjop70066-bib-0059] Turn on the Subtitles . (n.d.‐d). Let's use the summer holidays to improve children's reading. https://turnonthesubtitles.org/helping‐to‐avoid‐the‐summer‐slump/

[bjop70066-bib-0060] Vanderplank, R. (2016). Captioned media in foreign language learning and teaching: Subtitles for the deaf and hard‐of‐hearing as tools for language learning. Springer.

[bjop70066-bib-0061] Wexler, N. (2020). An easy way to help kids learn at home: Turn on the captions. *Forbes*. https://www.forbes.com/sites/nataliewexler/2020/04/18/an‐easy‐way‐to‐help‐kids‐learn‐at‐home‐turn‐on‐the‐captions/

[bjop70066-bib-0062] Wexler, N. (2021). Simple steps families can take to help kids with reading. *Forbes*. https://www.forbes.com/sites/nataliewexler/2021/12/02/simple‐steps‐families‐can‐take‐to‐help‐kids‐with‐reading/

[bjop70066-bib-0063] World Bank . (2019). Ending learning poverty: What will it take? The World Bank. https://documents1.worldbank.org/curated/en/395151571251399043/pdf/Ending‐Learning‐Poverty‐What‐Will‐It‐Take.pdf

[bjop70066-bib-0064] World Literacy Foundation . (2023). Turn on the subtitles. World Literacy Foundation. https://worldliteracyfoundation.org/turn‐on‐the‐subtitles/

[bjop70066-bib-0065] Yoon, J. O. , & Kim, M. (2011). The effects of captions on deaf students' content comprehension, cognitive load, and motivation in online learning. American Annals of the Deaf, 156(3), 283–289. 10.1353/aad.2011.0026 21941878

