# Supplementary material for: No evidence that same‐language subtitles improve children's reading fluency
Source: Br J Psychol. 2026 Mar 8;117(4):1308–25. doi: 10.1111/bjop.70066 (PMC13655959; doi:10.1111/bjop.70066)
Supplement: Supplementary file 1 [file BJOP-117-1308-s001.docx]

**Figure S1**. The illustration of the main scene and the subtitle regions in the eye-tracking experiment. The main scene region took an area of 1920 (the whole screen width) x 860 pixels and started from the top of the display screen. The subtitle region took an area of 1060 x 160 pixels and started right below the main scene region. It was symmetrical and started 430 pixels from the left side and ended 430 pixels to the right side of the screen.

**Table S1**. Preregistration deviations

| **No.** | **Type** | **Original wording** | **Deviation description** |
| --- | --- | --- | --- |
| 1 | Sample size:  Analysed fewer participants than planned | These factors were balanced in our plans to recruit 132 participants, 66 in the experimental and 66 in the control group. | Following the preregistered sample size, we recruited 132 children (65 girls and 67 boys) <...> Five children did not complete the intervention and had to be excluded from the study. In total, 127 children were included in the analysis: 64 children in the experimental group and 63 in the control group. Due to the length of the intervention it was not feasible to replace these participants. |
| 2 | Research question: Added a research question and exploratory analyses. | n/a | We included an exploratory analysis of the amount of intervention on the eye-tracking (Table S4) and reading fluency measures (Table S5). |
| 3 | Research question: Removed a qualitative analysis | In the experimental group only, we will contact the parents six weeks after the post-test to discuss whether exposure to subtitles had a long-lasting effect on the child’s reading and television viewing habits. We will report qualitative insights from this survey. | In the experimental group, only 53% of participants completed the six-week questionnaire. As a result, we decided to omit the corresponding qualitative analysis from the paper. |

**Table S2**. Summaries of LMMs that estimate the effects of group (experimental or control), session (pretest or posttest), and their interaction on eye-movement measures in children. Significant effects are shown in bold font.

|  | **Fixations**  **in the subtitle region** | | | | **Fixation durations**  **in the subtitle region** | | | | **Crossovers** | | | | **Skipped subtitles** | | | | **Skipped words** | | | |
| --- | --- | --- | --- | --- | --- | --- | --- | --- | --- | --- | --- | --- | --- | --- | --- | --- | --- | --- | --- | --- |
| *Predictors* | *Est.* | *SE* | *t* | *p* | *Est.* | *SE* | *t* | *p* | *Est.* | *SE* | *t* | *p* | *Est.* | *SE* | *t* | *p* | *Est.* | *SE* | *t* | *p* |
| (Intercept) | 0.38 | 0.03 | 12.42 | **<0.001** | 0.37 | 0.03 | 11.37 | **<0.001** | 0.22 | 0.01 | 19.05 | **<0.001** | 0.30 | 0.04 | 7.86 | **<0.001** | 0.60 | 0.02 | 39.72 | **<0.001** |
| Group (experimental) | -0.01 | 0.01 | -0.40 | 0.691 | -0.01 | 0.01 | -0.49 | 0.624 | -0.00 | 0.01 | -0.42 | 0.674 | 0.00 | 0.02 | 0.25 | 0.804 | 0.01 | 0.01 | 1.54 | 0.124 |
| Session (posttest) | 0.00 | 0.00 | 0.85 | 0.397 | 0.00 | 0.00 | 0.41 | 0.680 | 0.01 | 0.00 | 3.68 | **<0.001** | -0.01 | 0.01 | -2.39 | **0.017** | 0.00 | 0.00 | 0.25 | 0.805 |
| Group x Session | 0.00 | 0.00 | 0.42 | 0.672 | 0.00 | 0.00 | 0.48 | 0.633 | 0.00 | 0.00 | 1.20 | 0.233 | -0.01 | 0.01 | -0.87 | 0.382 | -0.00 | 0.00 | -1.88 | 0.060 |
| **Random Effects** | | | | | | | | | | | | | | | | | | | | |
| σ^2^ | 0.00 | | | | 0.00 | | | | 0.00 | | | | 0.01 | | | | 0.00 | | | |
| τ_00_ | 0.02 _participant_ | | | | 0.02 _participant_ | | | | 0.00 _participant_ | | | | 0.05 _participant_ | | | | 0.01 _participant_ | | | |
|  | 0.00 _video_ | | | | 0.00 _video_ | | | | 0.00 _video_ | | | | 0.00 _video_ | | | | 0.00 _video_ | | | |
| τ_11_ | 0.00 _participant.session_ | | | | 0.00 _participant.session_ | | | | 0.00 _participant.session_ | | | | 0.00 _participant.session_ | | | | 0.00 _participant.session_ | | | |
| ρ_01_ | -0.29 _participant_ | | | | -0.36 _participant_ | | | | 0.04 _participant_ | | | | -0.14 _participant_ | | | | -0.32 _participant_ | | | |
| ICC | 0.87 | | | | 0.87 | | | | 0.85 | | | | 0.89 | | | | 0.86 | | | |
| N | 126 _participant_ | | | | 126 _participant_ | | | | 126 _participant_ | | | | 126 _participant_ | | | | 126 _participant_ | | | |
|  | 4 _video_ | | | | 4 _video_ | | | | 4 _video_ | | | | 4 _video_ | | | | 4 _video_ | | | |
| Observations | 504 | | | | 504 | | | | 504 | | | | 504 | | | | 502 | | | |
| Marginal R^2^ / Conditional R^2^ | 0.001 / 0.874 | | | | 0.002 / 0.867 | | | | 0.013 / 0.850 | | | | 0.005 / 0.889 | | | | 0.017 / 0.865 | | | |

**Table S3**. Summaries of LMMs that estimate the effects of group (experimental or control), session (pretest or posttest), and their interaction on tests of reading fluency in children. Significant effects are shown in bold font.

|  | **TOWRE** | | | | **YARC**, log-transformed | | | |
| --- | --- | --- | --- | --- | --- | --- | --- | --- |
| *Predictors* | *Est.* | *SE* | *t* | *p* | *Est.* | *SE* | *t* | *p* |
| (Intercept) | 81.37 | 1.96 | 41.59 | **<0.001** | 4.51 | 0.05 | 94.81 | **<0.001** |
| Group (experimental) | -1.89 | 1.96 | -0.97 | 0.335 | 0.03 | 0.05 | 0.73 | 0.468 |
| Session (posttest) | 1.47 | 0.34 | 4.30 | **<0.001** | -0.04 | 0.01 | -4.60 | **<0.001** |
| Group x Session | 0.65 | 0.34 | 1.89 | 0.060 | 0.00 | 0.01 | 0.00 | 0.999 |
| **Random Effects** | | | | | | | | |
| σ^2^ | 29.73 | | | | 0.01 | | | |
| τ_00_ | 471.14 _participant_ | | | | 0.28 _participant_ | | | |
| ICC | 0.94 | | | | 0.95 | | | |
| N | 127 _participant_ | | | | 127 _participant_ | | | |
| Observations | 254 | | | | 254 | | | |
| Marginal R^2^ / Conditional R^2^ | 0.012 / 0.941 | | | | 0.008 / 0.950 | | | |

**Table S4**. Summaries of LMMs that estimate the effects of group (experimental or control), session (pretest or posttest), number of intervention hours, and their interaction on eye-movement measures in children. Significant effects are shown in bold font.

|  | **Fixations**  **in the subtitle region** | | | | **Fixation durations**  **in the subtitle region** | | | | **Crossovers** | | | | **Skipped subtitles** | | | | **Skipped words** | | | |
| --- | --- | --- | --- | --- | --- | --- | --- | --- | --- | --- | --- | --- | --- | --- | --- | --- | --- | --- | --- | --- |
| *Predictors* | *Est.* | *SE* | *t* | *p* | *Est.* | *SE* | *t* | *p* | *Est.* | *SE* | *t* | *p* | *Est.* | *SE* | *t* | *p* | *Est.* | *SE* | *t* | *p* |
| (Intercept) | 0.38 | 0.03 | 12.44 | **<0.001** | 0.37 | 0.03 | 11.39 | **<0.001** | 0.22 | 0.01 | 19.07 | **<0.001** | 0.30 | 0.04 | 7.87 | **<0.001** | 0.60 | 0.02 | 39.79 | **<0.001** |
| Group (experimental) | -0.01 | 0.01 | -0.45 | 0.656 | -0.01 | 0.01 | -0.55 | 0.586 | -0.00 | 0.01 | -0.45 | 0.656 | 0.01 | 0.02 | 0.30 | 0.765 | 0.01 | 0.01 | 1.54 | 0.125 |
| Session (posttest) | 0.00 | 0.00 | 0.85 | 0.397 | 0.00 | 0.00 | 0.41 | 0.680 | 0.01 | 0.00 | 3.70 | **<0.001** | -0.01 | 0.01 | -2.39 | **0.017** | 0.00 | 0.00 | 0.29 | 0.770 |
| Intervention | 0.00 | 0.00 | 1.16 | 0.247 | 0.00 | 0.00 | 1.29 | 0.196 | 0.00 | 0.00 | 0.60 | 0.550 | -0.00 | 0.00 | -1.23 | 0.220 | 0.00 | 0.00 | 0.05 | 0.963 |
| Group x Session | 0.00 | 0.00 | 0.43 | 0.671 | 0.00 | 0.00 | 0.48 | 0.629 | 0.00 | 0.00 | 1.21 | 0.229 | -0.01 | 0.01 | -0.86 | 0.390 | -0.00 | 0.00 | -1.87 | 0.062 |
| Group x Intervention (TV hours) | -0.00 | 0.00 | -0.13 | 0.896 | -0.00 | 0.00 | -0.21 | 0.833 | -0.00 | 0.00 | -0.53 | 0.596 | 0.00 | 0.00 | 0.38 | 0.704 | -0.00 | 0.00 | -0.06 | 0.949 |
| Session x Intervention (TV hours) | -0.00 | 0.00 | -0.06 | 0.951 | -0.00 | 0.00 | -0.13 | 0.899 | -0.00 | 0.00 | -0.25 | 0.804 | -0.00 | 0.00 | -0.34 | 0.734 | -0.00 | 0.00 | -0.47 | 0.636 |
| Group x Session x Intervention (TV hours) | -0.00 | 0.00 | -0.02 | 0.982 | -0.00 | 0.00 | -0.00 | 0.996 | -0.00 | 0.00 | -0.40 | 0.686 | -0.00 | 0.00 | -0.01 | 0.994 | -0.00 | 0.00 | -1.06 | 0.290 |
| **Random Effects** | | | | | | | | | | | | | | | | | | | | |
| σ^2^ | 0.00 | | | | 0.00 | | | | 0.00 | | | | 0.01 | | | | 0.00 | | | |
| τ_00_ | 0.02 _participant_ | | | | 0.02 _participant_ | | | | 0.00 _participant_ | | | | 0.04 _participant_ | | | | 0.01 _participant_ | | | |
|  | 0.00 _video_ | | | | 0.00 _video_ | | | | 0.00 _video_ | | | | 0.00 _video_ | | | | 0.00 _video_ | | | |
| τ_11_ | 0.00 _participant.session_ | | | | 0.00 _participant.session_ | | | | 0.00 _participant.session_ | | | | 0.00 _participant.session_ | | | | 0.00 _participant.session_ | | | |
| ρ_01_ | -0.29 _participant_ | | | | -0.36 _participant_ | | | | 0.04 _participant_ | | | | -0.15 _participant_ | | | | -0.32 _participant_ | | | |
| ICC | 0.87 | | | | 0.87 | | | | 0.85 | | | | 0.89 | | | | 0.86 | | | |
| N | 126 _participant_ | | | | 126 _participant_ | | | | 126 _participant_ | | | | 126 _participant_ | | | | 126 _participant_ | | | |
|  | 4 _video_ | | | | 4 _video_ | | | | 4 _video_ | | | | 4 _video_ | | | | 4 _video_ | | | |
| Observations | 504 | | | | 504 | | | | 504 | | | | 504 | | | | 502 | | | |
| Marginal R^2^ / Conditional R^2^ | 0.010 / 0.874 | | | | 0.012 / 0.867 | | | | 0.017 / 0.850 | | | | 0.015 / 0.889 | | | | 0.018 / 0.865 | | | |

**Table S5**. Summaries of LMMs that estimate the effects of group (experimental or control), session (pretest or posttest), number of intervention hours, and their interaction on tests of reading fluency in children. Significant effects are shown in bold font.

|  | **TOWRE** | | | | **YARC**, log-transformed | | | |
| --- | --- | --- | --- | --- | --- | --- | --- | --- |
| *Predictors* | *Est.* | *SE* | *t* | *p* | *Est.* | *SE* | *t* | *p* |
| (Intercept) | 81.32 | 1.94 | 41.98 | **<0.001** | 4.51 | 0.05 | 95.30 | **<0.001** |
| Group (experimental) | -1.78 | 1.94 | -0.92 | 0.360 | 0.03 | 0.05 | 0.71 | 0.476 |
| Session (posttest) | 1.49 | 0.34 | 4.36 | **<0.001** | -0.03 | 0.01 | -4.60 | **<0.001** |
| Intervention | -0.10 | 0.07 | -1.49 | 0.137 | 0.00 | 0.00 | 0.41 | 0.679 |
| Group x Session | 0.65 | 0.34 | 1.90 | 0.058 | 0.00 | 0.01 | 0.04 | 0.967 |
| Group x Intervention (TV hours) | 0.04 | 0.07 | 0.64 | 0.524 | -0.00 | 0.00 | -1.09 | 0.278 |
| Session x Intervention (TV hours) | -0.00 | 0.01 | -0.21 | 0.832 | -0.00 | 0.00 | -1.05 | 0.294 |
| Group x Session x Intervention (TV hours) | -0.01 | 0.01 | -1.07 | 0.286 | -0.00 | 0.00 | -1.37 | 0.171 |
| **Random Effects** | | | | | | | | |
| σ^2^ | 29.46 | | | | 0.01 | | | |
| τ_00_ | 461.08 _participant_ | | | | 0.28 _participant_ | | | |
| ICC | 0.94 | | | | 0.95 | | | |
| N | 127 _participant_ | | | | 127 _participant_ | | | |
| Observations | 254 | | | | 254 | | | |
| Marginal R^2^ / Conditional R^2^ | 0.033 / 0.942 | | | | 0.019 / 0.951 | | | |
